# Role of Zinc Signaling in the Regulation of Mast Cell-, Basophil-, and T Cell-Mediated Allergic Responses

**DOI:** 10.1155/2018/5749120

**Published:** 2018-11-25

**Authors:** Keigo Nishida, Ryota Uchida

**Affiliations:** ^1^Laboratory of Immune Regulation, Graduate School of Pharmaceutical Sciences, Suzuka University of Medical Science, 3500-3 Minamitamagaki, Suzuka, Mie 513-8607, Japan; ^2^Laboratory of Immune Regulation, Faculty of Pharmaceutical Sciences, Suzuka University of Medical Science, 3500-3 Minamitamagaki-cho, Suzuka, Mie 513-8670, Japan

## Abstract

Zinc is essential for maintaining normal structure and physiological function of cells. Its deficiency causes growth retardation, immunodeficiency, and neuronal degeneration. Zinc homeostasis is tightly regulated by zinc transporters and metallothioneins that control zinc concentration and its distribution in individual cells and contributes to zinc signaling. The intracellular zinc signaling regulates immune reactions. Although many molecules involved in these processes have zinc-binding motifs, the molecular mechanisms and the role of zinc in immune responses have not been elucidated. We and others have demonstrated that zinc signaling plays diverse and specific roles *in vivo* and *in vitro* in studies using knockout mice lacking zinc transporter function and metallothionein function. In this review, we discuss the impact of zinc signaling focusing particularly on mast cell-, basophil-, and T cell-mediated inflammatory and allergic responses. We also describe zinc signaling dysregulation as a leading health problem in inflammatory disease and allergy.

## 1. Introduction

This review discusses our current understanding of the roles of zinc transporters and zinc in inflammatory diseases and allergy. The essential trace element zinc [1] functions as a neurotransmitter [2] and an intracellular signaling molecule [3–6]. Effects of zinc on the immune and nervous systems have been demonstrated both in *in vivo* and *in vitro* studies, and zinc concentration is a key factor regulating these effects [7, 8]. A number of studies have shown that the depletion of zinc leads to impaired immune function. Decreased natural killer cell-mediated cytotoxic activity, antibody-mediated responses, and host defenses against pathogens and tumors have been observed in zinc-deficient mice [9–11].

Zinc plays an essential role in maintaining the conformation and the activity of many enzymes, transcription factors, signaling molecules, and other related factors. On the other hand, zinc can be toxic at a high concentration and can induce apoptosis in T and B cells [12, 13]. Intracellular concentration and distribution of zinc is controlled by zinc transporters including the *Slc39/Zrt- and Irt-like protein* (*ZIP*) and *Slc30/Zn transporter* (*ZnT*) families, which increase and decrease intracellular zinc levels, respectively [14–17], and this regulation is mediated by zinc-binding molecules such as metallothioneins [18, 19]. Zinc also acts as an intracellular signaling molecule. Extracellular stimuli can affect the intracellular zinc level either by modulating the transcription of zinc transporter genes and zinc-binding molecules or via transcription-independent routes such as zinc wave [20–22]. We categorize the former as late zinc signaling and the latter as early zinc signaling [3, 23]. While many studies have shown that zinc is important to the immune system and that imbalance in zinc homeostasis leads to various disorders, how zinc homeostasis and signaling are regulated in immune cells or whether zinc transporters and metallothioneins are involved in immune cell function is not fully understood.

Here, we describe how zinc and its homeostasis and signaling affect biological events in inflammatory and allergic responses.

## 2. Role of Zinc in Mast Cell Function

Mast cells play important roles in allergic reactions such as anaphylaxis, asthma, and atopic dermatitis [24–26]. Activated mast cells secrete two classes of mediators. One class of mediators are preformed mediators stored in granules which are rapidly degranulated and secreted upon activation. The second class of mediators are cytokines and chemokines that are synthesized *de novo* and gradually secreted. These secreted molecules play leading roles in allergic inflammatory responses.

The zinc probe Zinquin has been used to determine the intracellular zinc level and distribution and to visualize distinct zinc pools in allergy-related cells. Mast cell granules intensely fluoresce with Zinquin [27]. Airway epithelial cells are also rich in zinc [28]. Zinc deficiency increases allergic eosinophilic inflammation, whereas dietary zinc supplementation alleviates the symptom [29]. Interestingly, zinc deficiency is a risk factor for the development of asthma [30, 31]. Also, high *SLC39A2/ZIP2* expression levels have been reported in the leukocytes of asthmatic infants [32]. These reports indicate that zinc is involved in the development of allergic diseases. However, the precise roles of zinc and zinc transporters in allergy-related cells have not been fully elucidated.

**Figure 1:**
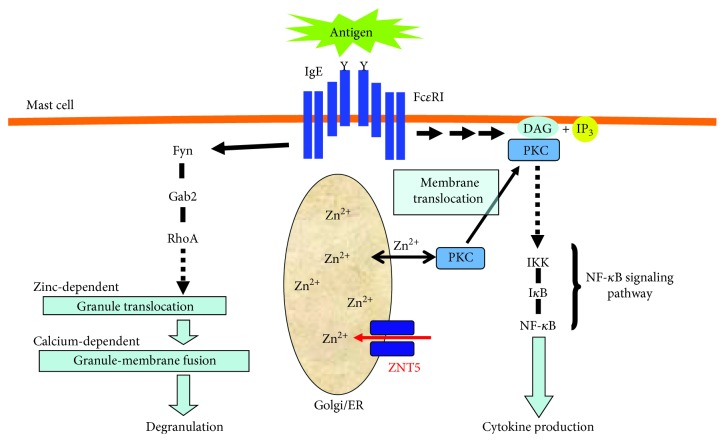
Zinc and zinc transporters are involved in Fc*ε*RΙ-mediated mast cell activation. Zinc is required in multiple steps of Fc*ε*RΙ-induced mast cell activation, including degranulation and cytokine production. Zinc levels depend on Fc*ε*RΙ-induced granule translocation, regulated by a Fyn/Gab2/RhoA-mediated signaling pathway. Zinc and ZNT5 are also required for PKC's translocation to the plasma membrane and NF-*κ*B's subsequent nuclear translocation, leading to the production of cytokines such as IL-6 and TNF*α*. IgE: immunoglobulin E; Fc*ε*RΙ: high-affinity receptor for IgE; Gab2: Grb2-associated binding 2; Zn: zinc; ZNT5: Zn transporter 5; ER: endoplasmic reticulum; PKC: protein kinase C; DAG: diacylglycerol; IP_3_: inositol-1,4,5-triphosphate; IKK: inhibitor of kappa B kinase; I*κ*B: inhibitor of kappa B; NF-*κ*B: nuclear factor-kappa B.

Zinc is required for both degranulation and cytokine production in mast cells [33] (Figure 1). The zinc chelator *N*,*N*,*N*′,*N*′-tetrakis(2-pyridylmethyl)ethylenediamine (TPEN) inhibits the release of histamine, the production of cytokines, and the secretion of lipid mediators in mast cells, and zinc supplementation rescues these inhibitory effects. Mast cell function is not affected by other metal chelators [33]. Similarly, zinc depletion caused by TPEN or the clinically used heavy metal chelator DMPS [34] inhibits the mRNA expression of chemokines such as eotaxin in human lung cell lines [35].

Mast cell degranulation begins when the stimulation of high-affinity receptor for IgE (Fc*ε*RΙ) triggers microtubule polymerization and granules are translocated to the plasma membrane. Fyn/Gab2/RhoA signaling, but not Lyn/SLP-76, plays a critical role in this calcium-independent and microtubule-dependent pathway [36]. However, in the second step of degranulation, which is calcium-dependent, the granules fuse with the plasma membrane. TPEN suppresses Fc*ε*RΙ-induced granule translocation, though it has little effect on calcium mobilization or other Fc*ε*RΙ functions such as the Fc*ε*RΙ-induced tyrosine phosphorylation of various signaling molecules. Since the translocation of granules depends on cytoskeletal proteins such as tubulin and actin [37] and microtubules are critical for both granule translocation and vesicle transport [36, 38], it was hypothesized that TPEN affects the microtubule assembly. However, as shown by Kabu et al., TPEN does not suppress Fc*ε*RΙ-induced microtubule formation, suggesting that its target might be zinc-regulated molecules that directly link microtubules and granules. Kinesin receptors are linker-cargo proteins essential for microtubule-dependent vesicle trafficking [39], and therefore, the target(s) of TPEN might interact indirectly with granules via kinesin.

TPEN suppresses Fc*ε*RΙ-mediated cytokine production as well as interleukin- (IL-) 6 and tumor necrosis factor- (TNF-) *α* mRNA transcription. Its stimulation activates protein kinase C (PKC), which is involved in cytokine production through nuclear factor-kappa B (NF-*κ*B) activation [40, 41]. Since TPEN inhibits the Fc*ε*RΙ-mediated translocation of PKC to the plasma membrane [33], PKC may be one of the targets of TPEN that affect cytokine production. In fact, PKC contains a zinc-binding motif and zinc is essential to maintain the structure of PKC [42]. Furthermore, its zinc-binding motif domain is required for the translocation of PKC to the plasma membrane after Fc*ε*RΙ stimulation [43].

*ZnT5* is highly expressed in mast cells, and Fc*ε*RΙ stimulation enhances its transcription level. *ZnT5*-KO mice have defects in mast cell-mediated delayed-type allergic reactions such as contact hypersensitivity, but not in immediate-type reactions such as anaphylaxis [44]. Consistent with these *in vivo* findings, *ZnT5* is required for Fc*ε*RΙ-mediated cytokine production, but not for mast cell degranulation.

Mast cells lacking *ZnT5* exhibit reduced levels of Fc*ε*RΙ-induced IL-6 and TNF-*α* mRNA, and ZNT5 is required for Fc*ε*RΙ-induced plasma membrane translocation of PKC and the nuclear translocation of NF-*κ*B [44]. Therefore, *ZnT5* is selectively required for mast cell-mediated delayed-type hypersensitivity reactions, and it is considered a novel component in PKC/NF-*κ*B signaling (Figure 1). Furthermore, it was shown in experiments using ZnT5-deficient DT40 cells that ZNT5 expressed on the ER-Golgi interface is necessary for the enzymatic activity of zinc-dependent alkaline phosphatases (ALPs) that are processed to the holo form between the ER and the Golgi [45–47]. Thus, ZNT5 may act to supply zinc to the zinc finger-like domains in PKC and ALP.

These findings show that zinc and its transporters are involved in the regulation of degranulation and cytokine production in mast cell-mediated allergic responses and that zinc transporters modulate the PKC/NF-*κ*B signaling pathway involved in the regulation of cytokine and chemokine gene expression.

## 3. Role of Zinc in Basophil-Mediated Cytokine Production

Basophils represent less than 1% of peripheral blood leukocytes. Like mast cells, they express Fc*ε*RΙ on the cell surface and release cytokines and chemical mediators in response to Fc*ε*RΙ activation [48, 49]. Under physiological conditions, basophils circulate in the blood while mast cells reside in peripheral tissues. Infiltration of basophils into peripheral tissues is often observed in allergic inflammatory diseases such as atopic dermatitis and bronchial asthma. Basophils have long been neglected in immunological studies due to their small numbers and morphological similarity to mast cells, but they are now recognized as a major source of cytokines such as IL-4 and TSLP that promote Th2-type allergic responses [50–53]. However, the molecular mechanisms of IL-4 production and the requirement of zinc in basophils have not been fully elucidated.

**Figure 2:**
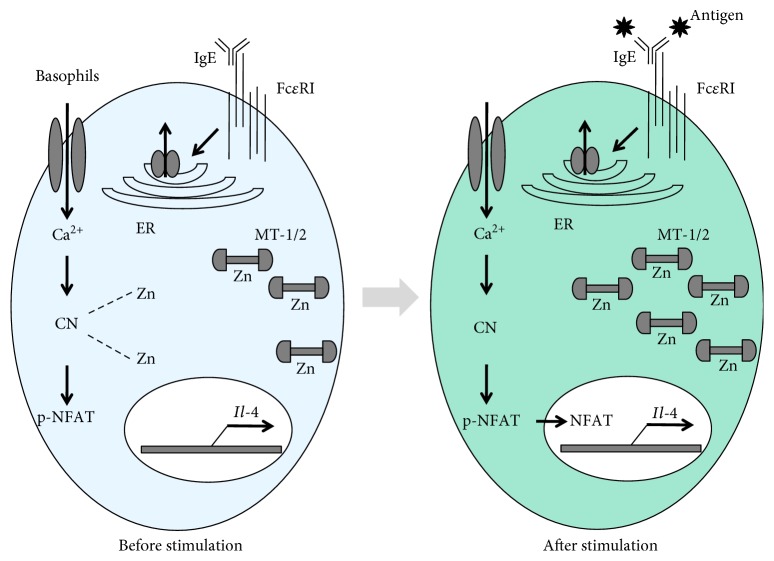
Metallothionein control in Fc*ε*RΙ-mediated IL-4 production in basophils. Fc*ε*RΙ stimulation activates basophils via signaling pathways that increase IL-4 production. Before stimulation, cytoplasmic free zinc ion inhibits CN. After Fc*ε*RΙ stimulation, cytoplasmic free zinc ion can induce the expression of metallothionein 1/2 and metallothioneins can bind to cytoplasmic free zinc ion. As a result, CN/NFAT signaling pathway can be activated and leads to increased IL-4 expression. IgE: immunoglobulin E; Fc*ε*RΙ: high-affinity receptor for IgE; ER: endoplasmic reticulum; Zn: zinc; MT-1/2: metallothionein 1/2; Ca: calcium; CN: calcineurin; NFAT: nuclear factor of activated T cells; IL-4: interleukin-4.

It has been reported that zinc-binding metallothionein (MT) proteins are required for Fc*ε*RΙ-induced IL-4 production in human and mouse basophils [54]. Transcription of *Mt-1* and *Mt-2* is significantly elevated after Fc*ε*RΙ stimulation in primary mouse basophils, while the expression of *Zips*, *ZnTs*, *Mt-3*, and *Mt-4* is not affected. Furthermore, Fc*ε*RΙ-induced IL-4 production in basophils was inhibited in the absence of *Mt-1* and *Mt-2*. It has also been shown that MTs are selectively required for Fc*ε*RΙ-induced calcineurin (CN)/nuclear factor of activated T cells (NFAT) signaling (Figure 2). Finally, Ugajin et al. indicated the requirement of MTs for IL-4 production by human basophils [55].

What is the role of free zinc in the regulation of IL-4 gene expression in basophils? CN consists of a catalytic subunit CN A and a regulatory subunit CN B, which form a heterodimer. The catalytic domain contains a Fe^3+^-Zn^2+^ dinuclear center, and zinc is thought to act as a catalytic cofactor [56, 57]. On the other hand, it has been reported that zinc inhibits the activity of CN *in vitro* [58–60]. The intracellular concentration of free zinc was higher in *Mt-1*/*2*-deficient basophils due to the absence of MTs, which play an important role in cytosolic zinc storage. Experimentally increased zinc levels attenuated the CN activity in basophils. These findings suggest that the regulation of intracellular zinc levels mediated by MTs plays an important role in the modulation of CN/NFAT signaling in basophils. Consistent with our reports, some researchers indicated zinc-dependent inhibition of cytokine expression in T cells [61–63]. Thus, we and the other group suggested that intracellular free zinc acts as a suppressor of cytokine production by the stimulation of immune cells. In contrast, zinc positively regulates the expression of cytokines. Zinc supplementation enhances the cytokine production in immune cells [64–66]. These conflicting effects may be due to concentration-dependent effect of zinc on different signaling molecules involved in cytokine expression.

## 4. Role of Zinc in T Cell Receptor-Mediated Signaling

Zinc signaling in T cells has been first described by Yu et al. (Ref. [67]). They observed an increase in intracellular zinc concentration within 1 minute after stimulation of the T cell receptor (TCR) [67]. This increase is dependent on the extracellular zinc concentration, suggesting that TCR stimulation induces an influx of extracellular zinc into T cells. Moreover, this influx of zinc is inhibited by silencing ZIP6, a zinc transporter expressed on the cytoplasmic membrane.

**Figure 3:**
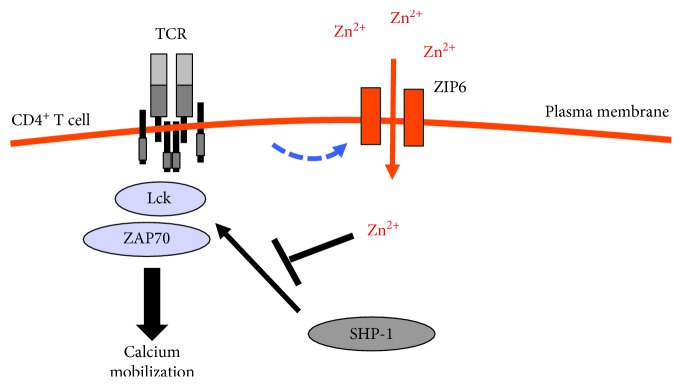
Zn functions as an ionic signaling molecule after T cell activation. Cytoplasmic zinc concentrations increased within 1 min after TCR triggering as the result of an influx via the zinc transporter *Zip6*. The increase was most pronounced in the immediate subsynaptic area and enhanced TCR signaling, at least in part as a result of the inhibition of SHP-1 recruitment. TCR: T cell receptor; Zn: zinc; SHP-1: Src homology region 2 (SH2) domain-containing phosphatase-1.

Early events in TCR signaling include tyrosine phosphorylation of several signaling molecules. The Src protein kinase Lck is primarily responsible for the phosphorylation of tyrosine residues within the ITAM motifs of CD3*ζ* and ZAP70 [68]. Extracellular zinc influences the phosphorylation of ZAP70 and inhibits negative regulatory feedback loops that at least partially accounts for the increase in ZAP70 phosphorylation. SHP-1, which dephosphorylates ZAP70 and other signaling molecules it is recruited to Lck [69], is a prime candidate target for ZIP6-mediated zinc signaling. In fact, the increase in zinc influx decreases the recruitment of SHP-1 to the TCR activation complex, augments ZAP70 phosphorylation, and sustains calcium influx. Yu et al. proposed that the influx of zinc following TCR stimulation leads to a local increase in cytoplasmic zinc that modifies early TCR signaling events. Thus, it is considered that ZIP6-mediated zinc signaling is dependent on extracellular zinc concentrations (Figure 3). In other words, extracellular zinc concentration controls the signal strength in TCR signaling. It has already been reported that the induction of IFN-*γ* production by zinc supplementation links the zinc status to Th1/Th2 polarization and corresponds to the observation that zinc deficiency leads to reduced Th1 immune response [70].

## 5. Role of Zinc in Inflammatory and Allergic Responses

The relationship between zinc homeostasis and immune function has been examined in a number of studies. The impact of single nutrient deficiency on immune response has been demonstrated by zinc homeostasis studies using experimental mouse and rat models. In addition, zinc deficiency is a frequent problem in humans and is associated with many chronic diseases. It is important to note that chronic diseases such as gastrointestinal disorders, chronic diarrhea, cirrhosis, renal disease, sickle cell anemia, some types of cancer, cystic fibrosis, pancreatic insufficiency, and autoimmune arthritis in humans can lead to a suboptimal zinc status [71–78]. Interestingly, zinc-containing compounds such as polaprezinc have been reported to improve the symptoms of autoimmune diseases in animal models [79, 80]. It has been suggested that the amelioration of autoimmune diseases by zinc occurs by inhibiting T cell activation, though detailed mechanisms involved in this process have not been elucidated.

Kitabayashi et al. hypothesized that zinc signaling might target proteins involved in inflammation and autoimmune diseases [81]. One such target protein is signal transducer and activator of transcription 3 (STAT3), which is a signaling molecule for the proinflammatory cytokine IL-6. The authors were particularly interested in how zinc affects the differentiation of Th17 cells that was known to be controlled by IL-6-induced STAT3 activation [82–85].

**Figure 4:**
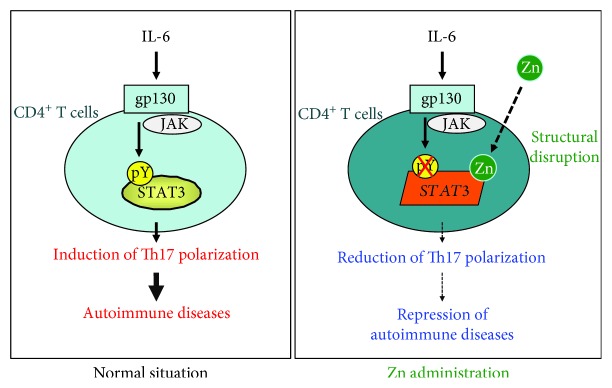
Zinc suppresses autoimmune diseases by inhibiting STAT3 activation. Zinc directly binds STAT3, altering its structure. The structurally altered STAT3 molecule cannot effectively transduce the IL-6 signaling pathway; this pathway is critical for autoimmune diseases involving Th17 cells. Zn: zinc; IL-6: interleukin-6; STAT3: signal transducer and activator of transcription 3; JAK: Janus kinases.

In addition, they used induced CIA (collagen-induced arthritis) and EAE (experimental autoimmune encephalomyelitis) mouse disease models, since both of these autoimmune diseases are mediated by antigen-specific Th17 cells and the development of these cells is controlled by STAT3 activation. Development of autoimmune diseases such as EAE was significantly suppressed by zinc supplementation. In one experiment, pathogenic Th17 cells were transferred into the mice and the mice were given zinc-supplemented drinking water. Development of EAE was similar in zinc-treated and control hosts, indicating that zinc supplementation did not alter the Th17 cell-mediated immune responses, including induced inflammation and Th17 cell activation. Thus, rather than affecting immune responses after pathogenic T cell development, zinc inhibits the development of pathogenic Th17 cells from naïve CD4^+^ T cells, a process that depends on the IL-6-STAT3 signaling pathway. In addition to this, Kitabayashi et al. demonstrated that zinc directly binds STAT3 and inhibits its phosphorylation by Janus kinases (JAK) and does so without affecting the kinase activity of JAK proteins. Furthermore, the structure of STAT3 itself is altered by the zinc binding [81] (Figure 4).

While zinc supplementation can help restore normal immune functions as described above, there are cases where chelating zinc can be beneficial to suppress antigen-dependent allergic responses. Kabu et al. reported that TPEN inhibited antigen-induced anaphylaxis response [33]. In this case, TPEN attenuated the degranulation of mast cells *in vivo* and serum histamine level was decreased in TPEN-treated mice. Fukuyama et al. reported that TPEN suppressed asthmatic responses in mouse models of ovalbumin- (OVA-) induced airway hyperresponsiveness and allergic airway inflammation [86]. TPEN also attenuated the upregulation of cytokines such as TNF*α*, IL-13, and IL-4 in bronchoalveolar lavage fluids and goblet cell hyperplasia after OVA exposure. These observations suggest that zinc chelators and their derivatives can be potential antiallergic drugs that act differently from currently available drugs such as histamine antagonists.

Zinc deficiency may enhance some allergic diseases. In some clinical studies, serum zinc levels were lower in children with atopic dermatitis (AD) compared to the control group [87, 88]. Interestingly, Kim et al. reported a correlation between AD and hair zinc level and reported that AD patients with a low hair zinc level showed clinical improvement after oral zinc supplementation [89]. Another case of zinc deficiency was reported. In this report, serum zinc levels were significantly lower in atopic asthmatics than nonatopic asthmatics and healthy controls. In atopic asthmatics, highly significant correlations were found between zinc levels and total IgE levels [90]. A small number of studies have been conducted to evaluate the efficacy of zinc supplementation to improve clinical symptoms of asthma during nonexacerbation [91, 92]. Thus, zinc supplementation might help patients with a low serum zinc concentration to recover from allergic diseases, but it must be well adjusted to the actual requirement. However, if zinc concentration significantly exceeds the physiological level, zinc may also adversely affect the immune function. The opposing effects of varying zinc concentrations on distinct signaling pathways in different cell types such as mast cells, basophils, and T cells remain to be investigated.

## 6. Conclusion and Perspectives

We have briefly reviewed recent findings showing that zinc contributes to allergy and autoimmune disorders. Zinc chelators effectively inhibit anaphylaxis, and zinc supplements may effectively suppress rheumatoid arthritis and multiple sclerosis, as indicated by studies using mouse disease models. These findings may lead to future therapeutic applications for suppressing inflammatory or allergic responses.

Using strategic gene targeting, we have demonstrated the role of zinc transporters and metallothioneins in various signaling pathways. Zinc therefore functions as an intracellular signaling molecule; its intracellular status is affected by extracellular stimuli regulating changes in zinc transporter and metallothionein expression. Much about the mechanism of zinc signaling remains to be clarified, including the method by which zinc transporters transfer zinc to individual target proteins such as PKC. We can be certain that further research will advance our understanding of zinc signaling and the intracellular transport of zinc in the immune system.
